# Viral RNA load as determined by cell culture as a management tool for discharge of SARS-CoV-2 patients from infectious disease wards

**DOI:** 10.1007/s10096-020-03913-9

**Published:** 2020-04-27

**Authors:** Bernard La Scola, Marion Le Bideau, Julien Andreani, Van Thuan Hoang, Clio Grimaldier, Philippe Colson, Philippe Gautret, Didier Raoult

**Affiliations:** 1 IHU-Méditerranée Infection, Marseille, France; 2 Aix Marseille Univ, IRD, APHM, MEPHI, Marseille, France; 3 Aix Marseille Univ, IRD, AP-HM, SSA, VITROME, Marseille, France; 4 Thai Binh University of Medicine and Pharmacy, Thai Binh, Viet Nam

**Keywords:** SARS-CoV2, Covid-19, RT-PCR, Co-culture, Viral load, Correlation

## Abstract

In a preliminary clinical study, we observed that the combination of hydroxychloroquine and azithromycin was effective against SARS-CoV-2 by shortening the duration of viral load in Covid-19 patients. It is of paramount importance to define when a treated patient can be considered as no longer contagious. Correlation between successful isolation of virus in cell culture and Ct value of quantitative RT-PCR targeting E gene suggests that patients with Ct above 33–34 using our RT-PCR system are not contagious and thus can be discharged from hospital care or strict confinement for non-hospitalized patients.

## Introduction

An outbreak of an emerging disease (Covid-19) due to SARS-CoV-2 started in Wuhan, China, then rapidly spread in China, and was declared pandemic on March 12, 2020, by the WHO [1–3]. Currently, the overall case fatality rate is about 2.3% in China, which is likely an overestimate because most patients have mild symptoms and are thus not tested [4]. Because of a study showing that chloroquine and hydroxychloroquine inhibit SARS-CoV-2 in vitro, we tested hydroxychloroquine as a treatment in Covid-19 patients [5, 6]. Our results show that in treated patients, the nasopharyngeal viral load of SARS-CoV-2-infected patients was cleared in only 3 to 6 days. Our results also suggest a synergistic effect of the combination of hydroxychloroquine and azithromycin, two molecules previously demonstrated to be active in vitro against Zika and Ebola viruses [7–10] and to prevent severe respiratory tract infections when administered to patients suffering viral infection [11]. These results are of great importance because a recent paper has shown that the median duration of viral RNA detection in patients suffering from Covid-19 in China was 20 days, with the longest duration being 37 days [12]. We are now facing a massive influx of patients in need of treatment and hospitalization, ideally in infectious disease wards equipped with NSB3 modules with negative pressure. Thus, in addition to obtaining complete disappearance of virus RNA in respiratory samples, having a PCR-based indicator of loss of contagiousness is a major priority for discharge from infectious diseases ward. Based on a set of 183 samples from 155 patients, we observed a significant relationship between viral RNA load and culture positivity.

The Méditerranée Infection University Hospital Institute in Marseille is the reference center for highly infectious diseases for Southeastern France. It was the only center in this region with diagnostic tests available during the first weeks of the epidemic in France and received patients’ samples from this whole area. From the day of the first positive test, on February 27 until March 12, 4384 clinical samples were tested by RT-PCR for 3466 patients. Since the beginning of this crisis and until now, on March 26, we inoculated 1049 samples and could obtain in culture 611 SARS-CoV-2 isolates. A total of 183 samples testing positive by RT-PCR, including 9 sputum samples and 174 nasopharyngeal swabs from 155 patients, were inoculated in cell cultures. SARS-CoV-2 RNA positivity in patient samples was assessed by real-time reverse transcription-PCR targeting the E gene, as previously described [13]. For all patients, 500 μL of nasopharyngeal swab fluid (Virocult, Elitech, France) or sputum sample were passed through 0.22-μm pore sized centrifugal filter (Merck Millipore, Darmstadt, Germany) and then were inoculated in 4 wells of 96-well culture microplates containing Vero E6 cells (ATCC CRL-1586) into Minimum Essential Medium culture medium with 4% fetal calf serum and 1% glutamine. All samples were inoculated between 4 and 10 h after sampling and kept at + 4 °C before processing. After centrifugation at 4000×*g*, microplates were incubated at 37 °C. They were observed daily for evidence of cytopathogenic effect. Two subcultures were performed weekly. Presumptive detection of virus in supernatant showing cytopathic effect was done using the SU5000 scanning electron microscope (Hitachi High-Tech Corporation, Tokyo, Japan) and then confirmed by specific RT-PCR targeting E gene. Variation of culture positivity rate was assessed statistically as the proportion of variance explained by Ct value and considered adequately fitted if the coefficient of determination (R^2^ statistic) was > 50%.

**Fig. 1:**
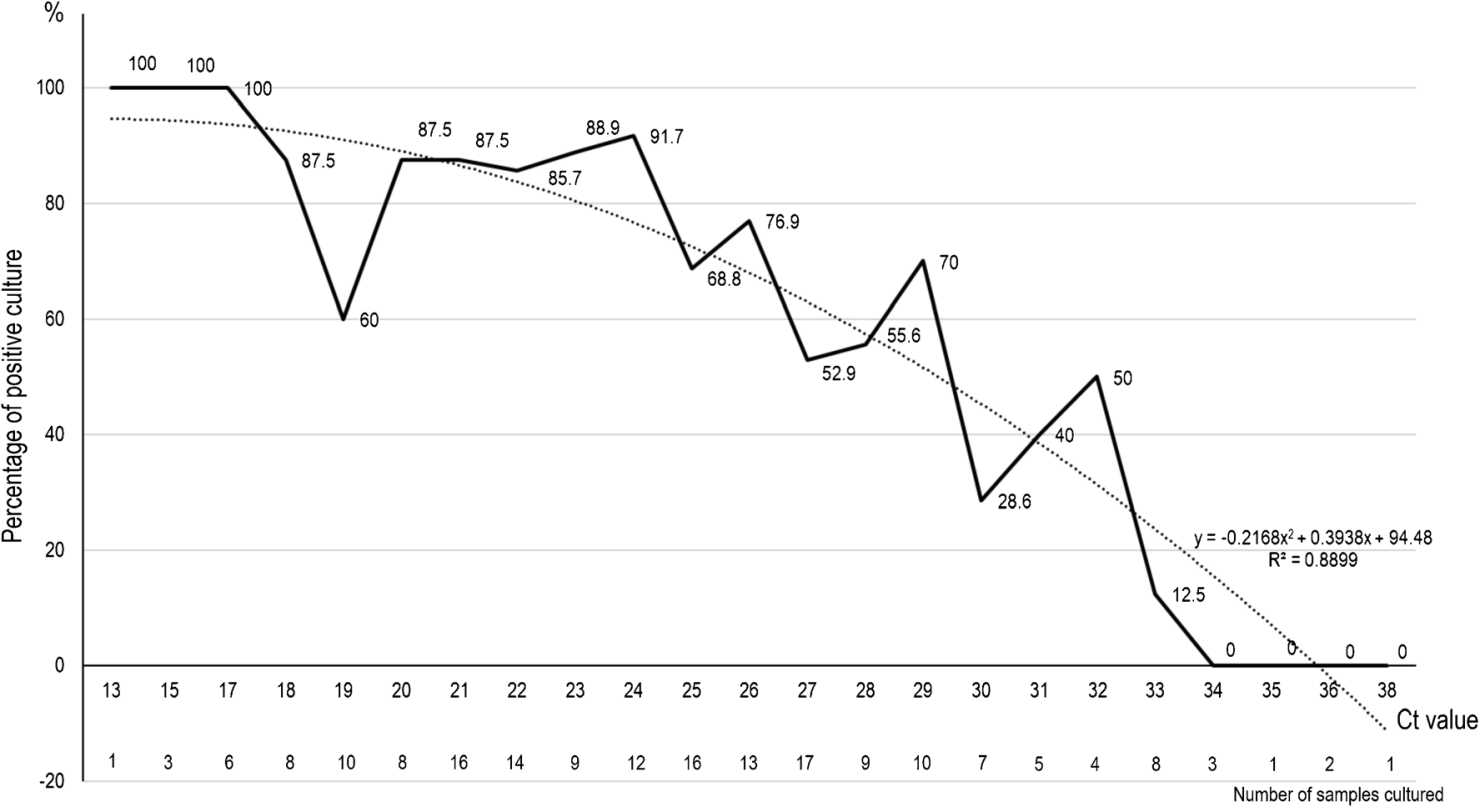
Percentage of positive viral culture of SARS-CoV-2 PCR-positive nasopharyngeal samples from Covid-19 patients, according to Ct value (plain line). The dashed curve indicates the polynomial regression curve

Among the 183 samples inoculated in the studied period of time, 129 led to virus isolation. Of these, 124 samples had detectable cytopathic effect between 24 and 96 h. Blind subcultures allowed obtaining 5 additional isolates only. We observed a significant relationship between Ct value and culture positivity rate (Fig. 1). Samples with Ct values of 13–17 all led to positive culture. Culture positivity rate then decreased progressively according to Ct values to reach 12% at 33 Ct. No culture was obtained from samples with Ct > 34. The 5 additional isolates obtained after blind subcultures had Ct between 27 and 34, thus consistent with low viable virus load.

In the present work, we observe a strong correlation between Ct value and sample infectivity in a cell culture model. On the basis of this data, we can deduce that with our system, patients with Ct values equal or above 34 do not excrete infectious viral particles. It was observed that SARS-CoV-2 was detected up to 20 days after onset of symptoms by PCR in infected patients but that the virus could not be isolated after day 8 in spite of ongoing high viral loads of approximately 10^5^ RNA copies/mL of sample, using the RT-PCR system used in the present study [14]. Progressive decrease of viral load over time is observed in all studies conducted in Covid-19 patients with positive detection being observed until 17–21 days after onset of symptoms, independently of symptoms [15]. These previous observations suggested that isolation of patients after diagnosis was mandatory. However, due to prolonged shedding of RNA in respiratory samples, the criteria for ending the isolation of a patient were not clear, and there was a need to correlate viral load to cultivable viruses. Our results show that in our system of RT-PCR, we can assess that patients with Ct equal or above 34 may be discharged. In 6 patients under the current therapeutic protocol used at our institute (hydroxychloroquine and azithromycin), Ct values > 34 were obtained between days 2 and 4 post-treatment [6]. There is no previous correlation demonstrated between level viral load in respiratory samples and infectivity. However, this reduction is the basis of all procedures used for the validation of disinfectants [16]. One limitation of our work is that it cannot be extrapolated to other hospital centers since they use different systems of sample transport, of RNA extraction, and of PCR with different primers and probes; i.e. it has been suggested that sensitivity of amplification based on Gene E detection would be less sensitive than ORF1ab or N genes. We propose that each center perform its own correlation between culture results and viral RNA load from patients’ samples. Another potential limitation is that nasopharyngeal swab fluid might be less representative than sputum samples. However, from the data obtained from patients rather tested in sputum, the viral load follows the same reduction with time of evolution than upper respiratory specimens [17].

## References

[1] Lai CC, Shih TP, Ko WC, Tang HJ, Hsueh PR (2020). Severe acute respiratory syndrome coronavirus 2 (SARS-CoV-2) and corona virus disease-2019 (COVID-19): the epidemic and the challenges. Int J Antimicrob Ag.

[2] Wang LS, Wang YR, Ye DW, Liu QQ (2020) A review of the 2019 novel coronavirus (COVID-19) based on current evidence. Int J Antimicrob Ag 105948. 10.1016/j.ijantimicag.2020.105948 10.1016/j.ijantimicag.2020.106137 PMC7434381 32826129

[3] WHO (2020) Director-General’s opening remarks at the media briefing on COVID-19 - 11 March 2020. https://www.who.int/dg/speeches/detail/who-director-general-s-opening-remarks-at-the-media-briefing-on-covid-19%2D%2D-11-march-2020

[4] Wu Z, McGoogan JM (2020) Characteristics of and important lessons from the coronavirus disease 2019 (COVID-19) outbreak in China: summary of a report of 72 314 cases from the Chinese Center for Disease Control and Prevention. JAMA 323. 10.1001/jama.2020.2648 10.1001/jama.2020.2648 32091533

[5] Yao X, Ye F, Zhang M, Cui C, Huang B, Niu P, et al (2020) In vitro antiviral activity and projection of optimized dosing design of hydroxychloroquine for the treatment of severe acute respiratory syndrome coronavirus 2 (SARS-CoV-2). Clin Infect Dis. 10.1093/cid/ciaa237 10.1093/cid/ciaa237 PMC7108130 32150618

[6] Gautret P, Lagier J-C, Parola P, Hoang VT, Meddeb L, Mailhe M, et al (2020) Hydroxychloroquine and azithromycin as a treatment of COVID-19: results of an open-label non-randomized clinical trial. Int J Antimicrob Ag 105949. 10.1016/j.ijantimicag.2020.105949 10.1016/j.ijantimicag.2020.105949 PMC7102549 32205204

[7] Retallack H, Lullo ED, Arias C, Knopp KA, Laurie MT, Sandoval-Espinosa C (2016). Zika virus cell tropism in the developing human brain and inhibition by azithromycin. Proc Natl Acad Sci.

[8] Madrid PB, Panchal RG, Warren TK, Shurtleff AC, Endsley AN, Green CE (2015). Evaluation of Ebola virus inhibitors for drug repurposing. ACS Infect Dis.

[9] Bosseboeuf E, Aubry M, Nhan T, de Pina JJ, Rolain JM, Raoult D (2018). Azithromycin inhibits the replication of Zika virus. J Antivirals Antiretrovir.

[10] Cao B, Parnell LA, Diamond MS, Mysorekar IU (2017). Inhibition of autophagy limits vertical transmission of Zika virus in pregnant mice. J Exp Med.

[11] Bacharier LB, Guilbert TW, Mauger DT, Boehmer S, Beigelman A, Fitzpatrick AM (2015). Early administration of azithromycin and prevention of severe lower respiratory tract illnesses in preschool children with a history of such illnesses: a randomized clinical trial. JAMA.

[12] Zhou F, Yu T, Du R, Fan G, Liu Y, Liu Z et al (2020) Clinical course and risk factors for mortality of adult inpatients with COVID-19 in Wuhan, China: a retrospective cohort study. Lancet. 10.1016/s0140-6736(20)30566-3 10.1016/S0140-6736(20)30566-3 PMC7270627 32171076

[13] Amrane S, Tissot-Dupont H, Doudier B, Eldin C, Hocquart M, Mailhe M, et al (2020) Rapid viral diagnosis and ambulatory management of suspected COVID-19 cases presenting at the infectious diseases referral hospital in Marseille, France, − January 31st to March 1st, 2020: a respiratory virus snapshot. Travel Med Infect Di 101632. 10.1016/j.tmaid.2020.101632 10.1016/j.tmaid.2020.101632 PMC7102626 32205269

[14] Woelfel R, Corman VM, Guggemos W, Seilmaier M, Zange S, Mueller MA, et al (2020) Clinical presentation and virological assessment of hospitalized cases of coronavirus disease 2019 in a travel-associated transmission cluster. Medrxiv. 10.1101/2020.03.05.20030502

[15] Zou L, Ruan F, Huang M, Liang L, Huang H, Hong Z (2020). SARS-CoV-2 viral load in upper respiratory specimens of infected patients. N Engl J Med.

[16] Kampf G, Todt D, Pfaender S, Steinmann E (2020). Persistence of coronaviruses on inanimate surfaces and its inactivation with biocidal agents. J Hosp Infect.

[17] Yu F, Yan L, Wang N, Yang S, Wang L, Tang Y et al (2020) Quantitative detection and viral load analysis of SARS-CoV-2 in infected patients. Clin Infect Dis. 10.1093/cid/ciaa345 10.1093/cid/ciaa345 PMC7184442 32221523

